# Topical Antiseptic or Antimicrobial Preparation for Oral Cavity Surgery: Is It Necessary and Does It Help?

**DOI:** 10.1002/lary.70273

**Published:** 2025-11-26

**Authors:** Veenadhari Kollipara, Kunal Koka, Christian Gluck, Neerav Goyal

**Affiliations:** ^1^ College of Medicine Pennsylvania State University Hershey Pennsylvania USA; ^2^ Department of Otolaryngology‐Head and Neck Surgery Penn State Hershey Medical Center Hershey Pennsylvania USA

## 1 Background

It is well established that the risk of surgical site infections (SSI) can be reduced with the use of topical and intravenous antiseptic and antimicrobial preparations for surgery requiring skin incisions [1]. However, there remains significant practice variability in using topical preparations for mucosal surgery in the head and neck [1]. As the oral cavity serves as an entryway to the aerodigestive tract, its diverse microbiome presents a unique challenge for infection prevention that differs from other surgical sites. For example, SSI prevention often includes the use of alcohol‐based antiseptics, which are contraindicated in head and neck and mucosal surgery [2]. Although general recommendations on topical antiseptic preparations exist, guidelines specific to head and neck surgery are still missing. This review will examine current evidence as to whether topical antiseptic and antimicrobial use in oral cavity surgery could lead to meaningful reduction in post‐operative morbidity.

## 2 Literature Review

Chlorhexidine gluconate (CHG) and povidine‐iodine (PI) are topical antiseptics which can be applied to mucosal surfaces preoperatively (before incision), intraoperatively (after incision but before closure), or postoperatively (after closure). Cetrimide, also known as alkyltrimethylammonium bromide, a lesser‐studied preoperative antiseptic, has been found to reduce intraoral bacterial counts and have long lasting effects [2]. Topical antimicrobials such as tetracycline and clindamycin represent an additional category that provides localized antimicrobial activity. There are a variety of described preparations, with preoperative mucosal preparation most frequently utilizing PI alone (17.9% of cases), followed by CHG alone (8.5%) and combination approaches using both agents (8.5%) [1].

A meta‐analysis of 4766 cases demonstrates that CHG application after oral surgery provides significantly better wound healing compared to controls (RR = 0.66, 95% CI = 0.55–0.80, *p* < 0.001), representing a 34% relative risk reduction in complications [3]. Laboratory testing confirms that 1% concentrations of CHG, PI, and cetrimide all achieve statistically significant bacterial reduction (*p* ≤ 0.001), with cetrimide being the most successful [2]. Additionally, specialized topical antimicrobials, including tetracycline ointment, have demonstrated specific efficacy with lower SSI rates compared to controls (OR 0.41, 95% CI 0.17–0.99) [2]. Furthermore, mouthwash interventions consistently identify CHG as the most frequently investigated antiseptic, with the majority finding no significant differences between CHG‐monotherapy and CHG combined with other products regarding gingival tissue healing over 7‐day periods [4].

A recent meta‐analysis of comparative studies involving 192 patients undergoing mucosal head and neck surgery in the oral cavity demonstrates that patients receiving topical antimicrobial prophylaxis have more than a 2‐fold decreased risk of SSI compared to those receiving systemic antimicrobials alone, with risk reductions ranging from 32% to 72% [5]. Localized prophylactic interventions substantially diminish infection rates when compared to systemic therapy alone. For example, an analysis of 554 patients undergoing upper aerodigestive tract reconstruction demonstrated that preoperative topical antiseptic mucosal preparation was associated with a significantly decreased risk of postoperative SSI (OR 0.49, 95% CI 0.30–0.77) [1]. This protective effect remained significant even after adjustment for confounding variables (OR 0.49, 95% CI 0.29–0.82) [1]. Microbiological analysis revealed that nearly 60% of infectious organisms included Pseudomonas, Klebsiella, and Staphylococcus species [1].

Data supports the use of antiseptics and antimicrobial topical preparations; however, there are challenges hindering the implementation of a standardized protocol. First, a shift in clinical practice would be necessary since preoperative and postoperative topical preparations are used only 30%–50% of the time [1]. Additionally, systematic reviews also reveal considerable heterogeneity across studies (*I* ^2^ = 85.9%), reflecting variation in the application methods, concentrations, and timing protocols (Table 1) [3]. Also, single applications may have a limited duration of effectiveness and repeated application of topical antiseptics may be necessary to maintain sustained antimicrobial benefit [2].

**TABLE 1:** Topical antiseptic and antimicrobial preparation examples for oral cavity surgery.

Topical preparation	Concentrations studied	Application timing studied
Antiseptics		
CHG	2.0%, 4.0% as aqueous [2] 0.006%—1% as a gel or a rinse [4] 0.12%, 0.2% [5]	Preoperative Postoperative
CHG combinations	CHG + antibiotics; CHG + chitosan [4] 0.2% CHG + anti‐discoloration system (ADS); 0.2% hyaluronic acid +0.2% CHG + ADS; 0.12% CHG + alcohol; 0.12% CHG + 0.5% α‐bisabolol^5^	Postoperative
PI	1%, 5%, 10% [2]	Preoperative Intraoperative
CHG‐PI	5% PVP‐I + 0.12% CHG [2] 10% PVP‐1 + 0.12% CHG [2]	Preoperative Intraoperative
Cetrimide	1% [2]	Preoperative
Antimicrobials		
Ampicillin‐carbenicillin	Applied as a powder [3]	Intraoperative
Clindamycin	Mouthwash [2]	Preoperative Postoperative
Piperacillin‐tazobactam	Mouthwash [3]	Intraoperative Postoperative
Tetracycline	3% Ointment [3]	Postoperative
Neomycin‐erythromycin and wintergreen mouthwash	Mouthwash [3]	Preoperative

*Note:* Preoperative—before incision, Intraoperative—after incision but before closure, Postoperative—after closure.

## 3 Best Practice

Topical antiseptic and antimicrobial preparations should be routinely implemented in oral cavity surgery, as patients receiving topical prophylaxis demonstrate more than a 2‐fold decreased risk of surgical site infections compared to systemic antimicrobials alone [5]. Most oral cavity procedures involving mucosal surfaces should incorporate aqueous‐based chlorhexidine gluconate, povidone‐iodine, or combination antiseptics as standard practice before an incision is made, with consideration for using topical antimicrobials for high‐risk procedures [2]. However, while current evidence strongly supports the routine use of topical preparations to reduce postoperative morbidity, further research on optimal selection, concentration, timing, and application methods remains necessary to develop standardized protocols given the considerable heterogeneity across studies [3].

## 4 Level of Evidence

These recommendations are based predominantly on Level 1 evidence from multiple systematic reviews and meta‐analyses, supported by Level 2 evidence from large prospective cohort studies and Level 4 evidence from observational studies.

## Funding

The authors have nothing to report.

## Conflicts of Interest

The authors declare no conflicts of interest.
